# Failure of Polyethylene Inlays in Cementless Total Hip Arthroplasty: A Retrieval Analysis

**DOI:** 10.1155/2016/5496396

**Published:** 2016-08-31

**Authors:** Ulrike Mueller, Christoph Lee, Christian Heisel, Marc Thomsen, Rudi G. Bitsch, J. Philippe Kretzer

**Affiliations:** ^1^Laboratory of Biomechanics and Implant Research, Clinic for Orthopedics and Trauma Surgery, Heidelberg University Hospital, Schlierbacher Landstrasse 200a, 69118 Heidelberg, Germany; ^2^Rehazentrum Bergmannstraße, Bergmannstr. 5, 10961 Berlin, Germany; ^3^Orthopädie Kurpfalz Speyer, Bahnhofstr. 51, 67346 Speyer, Germany; ^4^Klinikum Mittelbaden Baden-Baden Balg, Balger Str. 50, 76532 Baden-Baden, Germany

## Abstract

A retrieval analysis has been performed on 50 polyethylene inlays of cementless screw ring implants (Mecring, Mecron, Berlin, Germany) to investigate the failure mechanism of this specific open cup hip arthroplasty design that has shown a high clinical failure rate. Design-specific damage modes like rim creep, collar fatigue, and backside wear were assessed. Furthermore, the inlays were measured using a CMM to determine deformation. In 90% backside wear was observed and collar fatigue occurred in 68% of the cases. Rim creep was present in 38% of the polyethylene inlays. In 90% of the cases the cup opening diameter was 32.1 mm or less and 46% had a diameter less than 32 mm. It seems that creep and deformation of the polyethylene leads to a reduced diameter at the cup opening and consequently decreased clearance. To avoid this type of failure, polyethylene inlays should be supported at the back by the cup to reduce the risk of ongoing creep deformation.

## 1. Introduction

In total hip arthroplasty cementless cups were introduced into the European and North American markets in the early 1980s to overcome the high failure rates of cemented acetabular components in young and active patients.

The Mecring (Mecron, Berlin, Germany) was introduced as a cementless threaded cup, made of titanium alloy with a relatively smooth surface. It was designed as a metal ring open at the back of the cup. The polyethylene (PE) inlay was fixed in the ring using a snap-fit mechanism additionally supported by a collar.

After encouraging early clinical results [1] high failure rates became obvious in the middle and long term [1–10]. The cup frequently showed migration, instability, and tilting which consequently lead to aseptic loosening [1–3, 5–7, 9, 10]. Clinical studies showed revision rates due to aseptic loosening of 35% after 14 years [2] and more than 50% after 17 years [6]. Such implant failures may be related to polyethylene wear, missing primary stability, surgical preparation, cup positioning, surface structure, postoperative loading, and the pattern of mechanical stress distribution within the implant-bone-interface potentially leading to stress shielding during functional loading [11, 12].

In this study a retrieval analysis has been performed on the polyethylene inlays aiming to assess potential failure mechanisms related to the specific design of the Mecring. It was suggested that the polyethylene inlay is unfavourable supported by the cup leading to creep and deformation and consequentially to narrowing of the cup opening due to the clinical use.

## 2. Materials and Methods

For the consecutive retrieval analysis 55 Mecring components consisting of the cup and the polyethylene inlay were available. Five components were excluded because two of them were heavily damaged during the explantation and in three cases all clinical data was not available. In all cases, the reason for revision surgery was aseptic loosening. Patient demographics and implant related data are given in Table 1.

To evaluate the material deterioration a qualitative damage assessment was performed followed by geometric measurements of the components.

**Figure 1:**
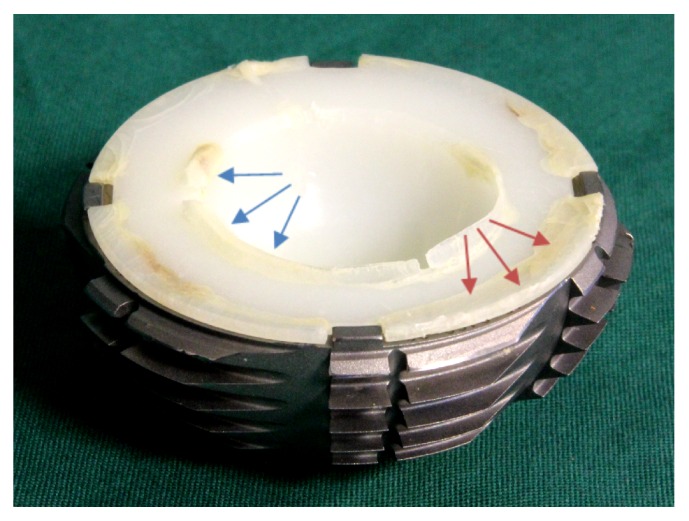
Examples for the design-specific damage modes: rim creep at the cup opening (blue arrows) and collar fatigue at the flanges on the outer rim (red arrows).

**Figure 2:**
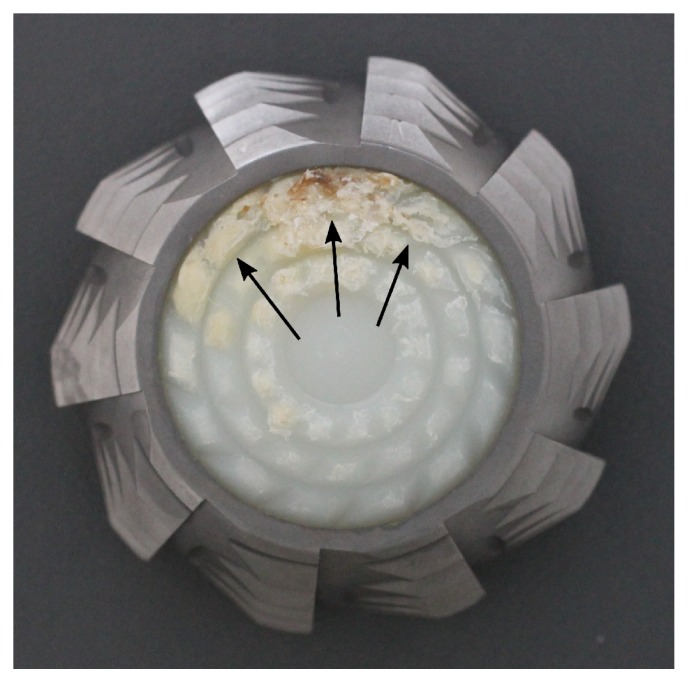
Example for backside wear at the underside of the inlays (black arrows).

The retrieved polyethylene inlays were visually examined for the evidence of damage or alterations. Three major parameters were identified:

1. Deformation and fatigue at the collar of the polyethylene inlay in the area, where the collar (outer rim) is in contact with the titanium acetabular shell: this has been defined as* collar fatigue*. An example is given in Figure 1 (red arrows).
2. Creep and deformation at the inner rim of the polyethylene inlay, leading to narrowing at the cup opening: this has been defined as* rim creep*. An example is given in Figure 1 (blue arrows).
3. Wear at the protruding back of the polyethylene inlay: this has been defined as* backside wear*. An example is given in Figure 2.

These three signs were graded depending on the severeness and extent of the damage by two independent observers (UM, JPK). Hereby the extent was graded on a 0–5 scale. A score of 0 means that no damage was detected at the corresponding region of the polyethylene liner. The score 1 corresponds to less than 20% of the surface area and the score 5 to more than 80%. Hereby, the extent of the damage was evaluated along with the severeness on a 0 (none) to 5 (severe) scale. Both numbers were added to calculate a combined score (0 to 10). The interrater reliability between both observers has been evaluated using Kappa statistics and the average score of both was used for the final score. Furthermore, the intrarater reliability has been calculated based on 15 samples for one observer.

For some components it was noticed that the head moved easily in the polyethylene insert, whereas others got stuck in it. To evaluate this effect, the equatorial diameter of the retrieved inserts was measured optically based on an edge detection algorithm, using a 3-dimensional coordinate measuring machine (Mahr, Multisensor, MS 222, Goettingen, Germany). The CMM was accurate within ±3 *µ*m. The equatorial diameter of the inlays was measured at the cup opening. Each polyethylene inlay was measured three times and the mean was calculated.

**Figure 3:**
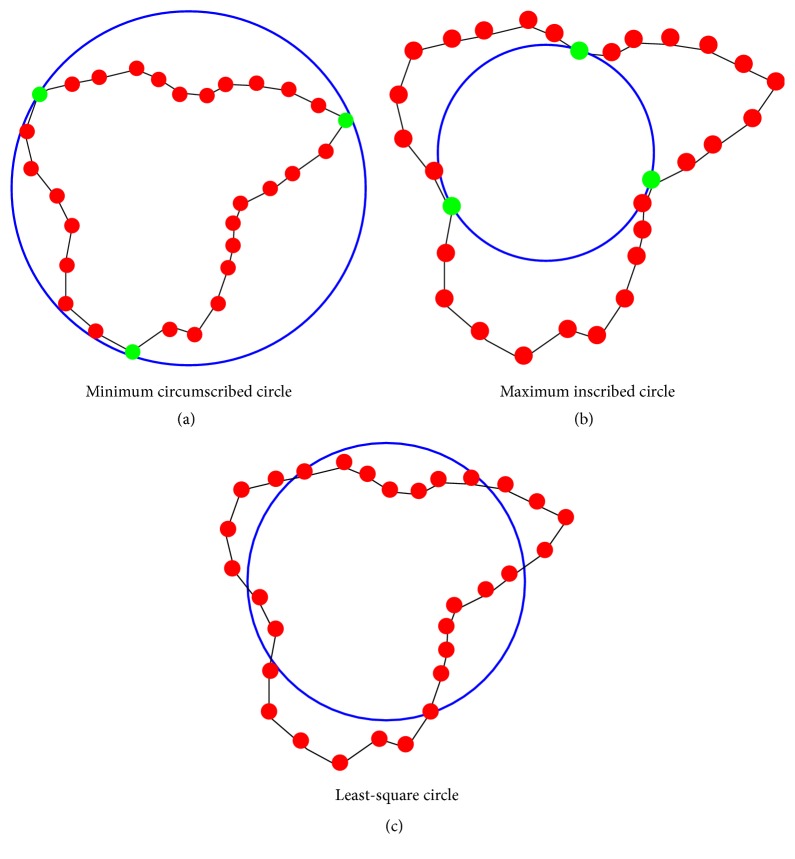
The three circle approximation methods to calculate the equatorial diameter.

To determine the equatorial diameter three different circle approximation methods are available: the minimum circumscribed circle (MCC), the maximum inscribed circle (MIC), and the least-square circle (LSC) (Figure 3).

The minimum circumscribed circle (MCC) is defined as the smallest circle which encloses all measuring points of the measured profile (all measuring points are in the inside of the circle; see Figure 3(a)).

The maximum inscribed circle (MIC) is defined as the largest circle which fits in the measuring profile (all measuring points are outside the circle; see Figure 3(b)).

The least-square circle (LSC) determines a best fit circle that is located mostly in the middle between the measuring points (Figure 3(c)).

The observed motion inhibition of the head in the polyethylene inlay was assumed to be related to deformation at the inner rim of the inlay. Because the MIC represents the smallest possible equatorial diameter, it has been chosen as most relevant for further analysis.

To consider the clinical data the damage scores were correlated to time to revision and BMI using Spearman's correlation. The diameter of the cup opening was also correlated to time to revision and BMI. The cup opening diameter was classified into four groups and a group-wise comparison was performed for each damage score using Kruskal-Wallis test.

## 3. Results

### 3.1. Reliability

Cohen's kappa statistic revealed agreement between both observers (interrater reliability) in any case (*p* < 0.005). Substantial strength of agreement was found for backside wear (*κ* = 0.755), whereas the agreement for rim creep (*κ* = 0.593), collar fatigue (*κ* = 0.570), and total damage (*κ* = 0.420) was moderate. The intrarater reliability also showed moderate to substantial agreement (*κ* = 0.442–0.840, *p* < 0.005).

### 3.2. Damage Scores

**Figure 4:**
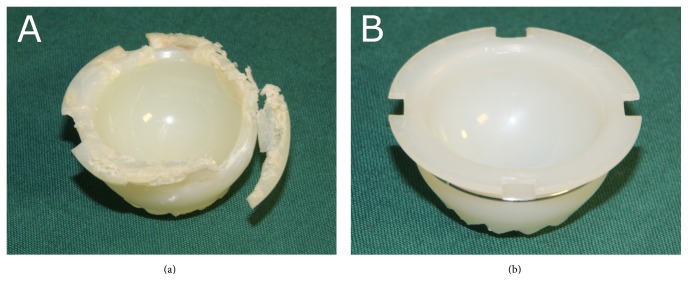
Two examples of the polyethylene inlays: severe damage in terms of backside wear, rim creep, and collar fatigue is obvious after 18.5 years in situ (a) and a mild case whereas only minimal backside wear occurred after 7.6 years in situ (b).

**Figure 5:**
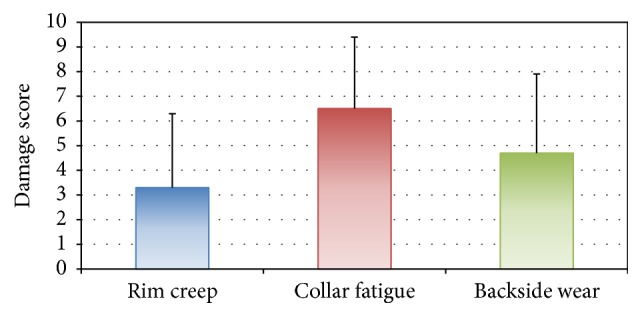
The scores are given for each type of damage. The mean and the standard deviation are shown.

Rim creep was present in 38% of the polyethylene inlays, whereas backside wear was seen in 90% of the inlays and collar fatigue occurred in 68% of the cases. The total damage score ranged from 1 to 27 (14.5 ± 7.2). Examples for a severe damaged inlay (damage score = 26.5) and an inlay with low damage (damage score = 1) are shown in Figure 4. The assessed damage scores for each type of damage are given in Figure 5.

### 3.3. CMM

In 11 cases CMM measurements were not feasible because the inner rim of the cup was widely damaged. Therefore 39 of 50 retrieved inlays were included in CMM analysis.

**Figure 6:**
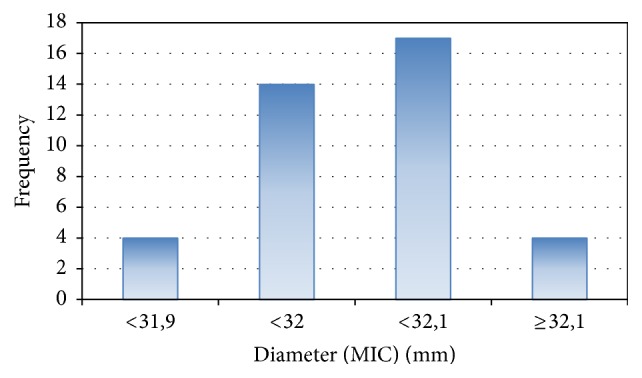
Distribution of the cup opening diameter of the 39 inlays.

Depending on the analytical approach the diameters varied between 31.997 ± 0.127 mm (MIC), 32.166 ± 0.163 mm (LSC), and 32.334 ± 0.253 mm (MCC). Regarding the MIC diameter, 35 of the 39 inlays (90%) had a diameter of 32.1 mm or less, and 18 inlays (46%) had a diameter less than 32 mm (Figure 6).

Rim creep and backside wear showed a weak but significant correlation to time to revision. No correlation was found for the BMI and the damage scores (Table 2). Also, the cup opening diameter did not correlate to the time to revision (*R* = 0.103, *p* = 0.532) and to BMI (*R* = 0.183, *p* = 0.270).

**Figure 7:**
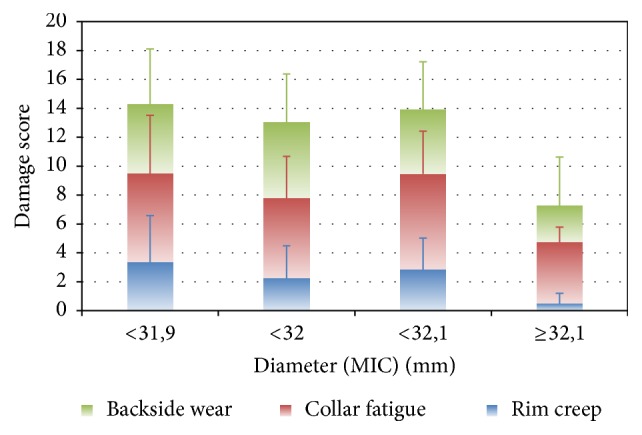
Comparison of the damage scores depending on the cup opening diameter. The total height of the bars corresponds to the total damage score. The mean values and the standard deviations are shown.

In Figure 7 the damage scores are compared depending on the cup opening diameter. Lower damage scores are obvious if the cup opening is equal to or greater than 32.1 mm. For rim creep this difference was statistically significant (*p* = 0.008).

## 4. Discussion

The Mecring was a popular first generation uncemented, threaded cup for arthroplasty of the hip. But this implant showed unacceptably high failure rates in the middle and long term [1–10].

This retrieval analysis on 55 failed Mecring components revealed different findings. Creep, fatigue, and backside wear were frequently observed and increased over time. These are typically findings for polyethylene degradation in joint replacement [13–15]. However, the specific localization and type of damage support the suggestion that the implant design takes part in the damage formation and failure of the implants. It was observed that the clearance between head and inlay was too small in many cases. This becomes obvious, firstly because several heads were hard to rotate in the polyethylene inserts by hand and secondly because the CMM measurements revealed that the cup opening diameter was frequently below the head diameter. In 90 percent of cases, the cup opening diameter was smaller than 32.1 mm, whereas in the ISO 7206-2 a clearance of 0.1 to 0.3 mm is recommended for polyethylene inserts [16].

**Figure 8:**
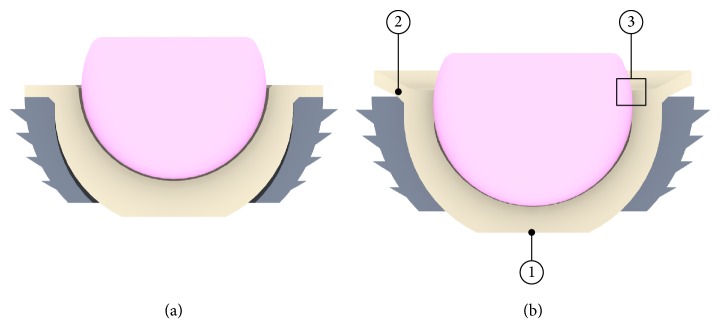
Assumed failure mechanism of the Mecring: the correct function of the PE inlay in the cup as initial state is shown in (a) whereas (b) shows the deformed inlay with narrowing at the inner rim and backside wear caused by cold flow of PE.

The following mechanism may explain these observations: it is assumed that the inlays were originally manufactured with a sufficient clearance that allows free articulation of the head in the insert. Thus, the inlay geometry might have changed over time in situ. This geometrical alteration is related to a missing support on the back of the inlay and overloading of the collar over time. Initially the inlay is well fixed based on the snap-fit mechanism and the collar is equally supported by the cup (Figure 8(a)). Due to in vivo loading the polyethylene begins to creep and this causes a flow of the material into the cup. As the cup is open, creep will not be limited to a certain extent. Consequently, the polyethylene gets into contact with the bone behind the cup and backside wear may occur (Figure 8(b)(1)). Simultaneously, increased stresses are acting at the back of the collar leading to deformation and fatigue (Figure 8(b)(2)). In progress the deformation will lead to narrowing of the inner rim of the polyethylene inlay (Figure 8(b)(3)). This will result in a reduced diameter at the cup opening and decreased clearance. Increased friction between the head and the inlay will cause higher stresses at the bone implant interface and may contribute to loosening of the implant.

This assumption is supported by the observation that the damage scores were smaller if the cup opening diameter was above 32.1 mm (Figure 7).

Typically creep occurs within the first one or two years after implantation [17, 18]. If the polyethylene is not sufficiently supported at the back, creep may continue to occur. In this study, the analyzed retrievals have been in situ for at least three years and creep progression has been observed over time, although the correlation was not strong. Another possible explanation for the deformation and creep of the polyethylene could be that the screw ring was not stiff enough to withstand acetabular loading.

However, the described mechanism remains an assumption as several limitations have also to be considered. To exactly determine the creep deformation the original geometry of the inlays would have been essential. These data have not been available. Furthermore, the damage score grading has been subjective although good agreement between different observers was found. Regarding the damage, potential oxidation has not been quantified although it takes part in degradation process. The inner rim was frequently damaged. Therefore, an optical measurement method has been chosen to better assess the rim of the polyethylene inlay in comparison to a tactile method. However, in 11 cases the rim was severely damaged leading to excluding them for the measurements of cup opening diameter. Beside these limitations the relatively smooth surface of the cup has also been discussed as another reason for the high incidence of revisions [2, 10].

## 5. Conclusion

In conclusion, a polyethylene inlay should be supported at the back to avoid ongoing creep deformation. This is of particular importance if the inlay has a collar. The combination of both may compromise the joint articulation leading to failure of the implant which should be avoided.

## Figures and Tables

**Table 1:** Patient demographics and implant related data of the 50 Mecring components.

	Parameter	Value
Patient	Number of patients^*∗*^	49
	Age (at the time of implantation), in years	53 ± 12 (21–70)
	Age (at the time of revision surgery), in years	62 ± 13 (26–79)
	Time to revision, in years	9.1 ± 3.3 (3.0–18.5)
	Sex	
	Female	30 (61%)
	Male	19 (39%)
	Side	
	Left	27 (54%)
	Right	23 (46%)
	BMI, in kg/m^2^	26.4 ± 4.3 (17.7–35.3)

Implant	Design, *n* (%)	
	Mecring A (Type A)	42 (84%)
	Mecring B (Type B)^*∗∗*^	8 (16%)
	Cup size	46–62 mm
	Head size	32 mm in all cases
	Head material	
	Ceramic (BIOLOX forte)	45 (90%)
	CoCr	5 (10%)

^*∗*^One patient underwent bilateral hip replacement.

^*∗∗*^Type B is a newer design of the acetabular component with an increase in thread width and depth.

**Table 2:** Spearman correlation coefficients for the damage scores correlated to clinical data (*n* = 50).

	Time to revision		BMI	
	*R*	*p*	*R*	*p*
Rim creep	0.291	0.040	0.165	0.262
Collar fatigue	0.248	0.083	0.097	0.513
Backside wear	0.367	0.009	0.119	0.421
Total damage	0.359	0.011	0.183	0.212
